# Patients’ preferences for headache acute and preventive treatment

**DOI:** 10.1186/s10194-017-0813-3

**Published:** 2017-10-06

**Authors:** Dimos D. Mitsikostas, Ioanna Belesioti, Chryssa Arvaniti, Euthymia Mitropoulou, Christina Deligianni, Elina Kasioti, Theodoros Constantinidis, Manolis Dermitzakis, Michail Vikelis, on behalf of the Hellenic Headache Society

**Affiliations:** 1 First Neurology Department, Aeginition Hospital, School of Medicine, National & Kapodistrian University of Athens, 72-74 V. Sofias’ Avenue, 11528 Athens, Greece; 2 Second Neurology Department, Attikon Hospital, School of Medicine, National & Kapodistrian University of Athens, Athens, Greece; 3 Private Headache Clinic, Korinthos, Greece; 4 Department of Neurology, “Geniki Kliniki” Euromedica, Thessaloniki, Greece; 5 Headache Clinic, Mediterraneo Hospital, Glyfada, Greece

**Keywords:** Migraine, Tension-type headache, Cluster headache, Neurostimulation, Monoclonal antibodies, Patient-centeredness, Equity of healthcare, Nocebo

## Abstract

**Background:**

We aimed to explore patients’ preferences for headache treatments with a self-administered questionnaire including the Q-No questionnaire for nocebo.

**Methods:**

Questionnaires from 514 outpatients naïve to neurostimulation and monoclonal antibodies were collected.

**Results:**

Patients assessed that the efficacy of a treatment is more important than safety or route of administration. They preferred to use an external neurostimulation device for both acute (67.1%) and preventive treatment (62.8%). Most patients preferred to take a pill (86%) than any other drug given parenterally for symptomatic pharmaceutical treatment. For preventive pharmaceutical treatment, most patients preferred to take a pill once per day (52%) compared to an injection either subcutaneously or intravenously each month (9% and 4%), or three months (15% and 11%). 56.6% of all participants scored more than 15 in Q-No questionnaire indicating potential nocebo behaviors that contributed significantly in their choices.

**Conclusion:**

These patient preferences along with efficacy and safety data may help physicians better choose the right treatment for the right person.

## Background

Several agents with ever-newer mechanisms of action and neurostimulation techniques are testing for acute or preventive treatment of migraine and cluster headache developing an explosive therapeutic environment [1–3]. Apart from four injectable monoclonal anti-CGRP antibodies, new treatments include oral agents (CGRP antagonists, 5-HT1F agonists and mGlu5 receptor modulators) and several neurostimulation devices for both symptomatic and prophylactic treatment of migraine and cluster headache. Whether these treatments will be finally commercially available depends on the results of phase 3 clinical trials in progress [1–3]. New treatments target to improve efficacy, safety, tolerance and adherence. Among other factors, adherence is related to treatment efficacy, safety, tolerance, duration and route of administration; it is very poor in migraine. Only one out of four migraineurs comply with the current available treatments for chronic migraine when a treatment is required for six months; and only one out of five migraineurs comply when the duration of the preventive treatment increases up-to one year [4]. To improve adherence, the patients’ perspectives and preferences should be taken into account in the choice of treatment [5].

There are three general models for decision-making regarding medical treatment: the paternalistic model, the informed model, and the shared model [6]. The classic “paternalistic model” is one in which the physician makes medical decisions for the patient without substantial consideration of the patient’s preferences. The “informed model” involves the physician communicating information to the patient regarding treatment options, risks, and benefits. After being provided sufficient information, the patient ultimately makes an informed treatment decision based on their preferences. The “shared model” involves the physician discussing treatment options and preferences with the patient and then both parties actively participate in making a shared medical decision [6, 7]. Headache sufferers prefer the “shared model” approach to medical decision making in regards to the prescription of triptans [7], but the patients’ preferences for the preventive anti-migraine treatments have not been investigated so far. This study aimed to systematically and prospectively record patients’ preferences related to symptomatic and preventive treatments for migraine and other primary headache disorders in the context of patient-centered medicine. Since nocebo may affect patient choices, we also aimed to investigate this cofactor in our patient population by using the Q-No questionnaire [8].

## Methods

This is a project designed by the Hellenic Headache Society. Five outpatient headache centers in Athens participated in the survey. After explaining the scope of the survey, reaching an agreement and signing the associated consent form, the participants were invited to fulfill a self-administered questionnaire (maximum time 10 min). All questionnaires were collected and kept by the department nurse. The main questionnaire (questionnaire A) consisted of 11 questions (Appendix). To assess the internal consistency of the questionnaire, a second questionnaire (questionnaire B) included the same 11 items but rephrased, was delivered to 10% of participants after fulfilling the first one. To test the consistency of answers (test-retest reliability) the main questionnaire A was applied once again in another proportion of participants (10%), a month later. The Q-No questionnaire was included in the questions that have been addressed to patients (four additional questions). It is a self-fulfilled questionnaire that predicts potential nocebo behavior in outpatients seeking neurological consultation. The Q-No predicts nocebo with 71.7% specificity, 67.5% sensitivity and 42.5% positive predictive value [8]. The participants were consecutive outpatients seeking neurological consultation for their headaches. The inclusion criteria were: (i) both genders, age 18–65 years; (ii) diagnosis of any primary headache disorder according to IHC-IIIbeta [9]; (iii) current preventive pharmaceutical treatment for headache lasting for more than 3 months; (iv) other medical conditions and medication overuse were allowed; (v) patients should be able to understand the Greek language and signed a consent form. The participant professions were classified according to the International Standard Classification of Occupations (ISCO-08) [10] into eleven categories: unemployed, managers, professionals, technicians and associate professionals, clerical support workers, service and sales workers, skilled agricultural, forestry and fishery workers, craft and related trades workers, plant and machine operators, and assemblers, elementary occupations and armed forces occupations. The education of participants was classified according to the International Standard Classification of (ISCDE 2011) [11] into ten categories: less than primary, primary, lower secondary, upper secondary, post secondary non-tertiary, short-cycle tertiary, Bachelor’s or equivalent level, Master’s or equivalent level, Doctoral or equivalent level and not elsewhere classified. The ethical and the scientific committees of all five Headache Centers approved the study protocol and all patients signed a consent form.

### Statistics

Categorical variables are expressed in frequencies and percentages. Chi-square test with continuity correction was used to assess the association between the categorical variables (nominal or ordinal). The odds ratio applied in order to measure the magnitude of association. Associations between dependent variables and independent variables were analyzed using logistic regression model. Subgroup analyses were performed for the primary headache disorder; age; sex; frequency of headaches (episodic versus chronic types); education; occupation; and nocebo. All statistical tests were two-sided and *p* values of 0.05 or less were considered as statistically significant. Statistical analyses were conducted using the Software IBM-SPSS (Statistical Package for the Social Sciences -Version 24).

## Results

Questionnaires from 514 consecutive headache patients were collected during May and July 2016. Interestingly, no patient denied participating in the study. The descriptive demographics and the analysis of fulfilled questionnaires by primary headache disorder they were suffering from are summarized in Tables 1 and 2, respectively. Forty-two participants (8.17%) re-fulfilled the questionnaire A after a month without any difference from the initial one. Forty-nine participants (9.5%) reported the same answers as well in a rephrased questionnaire. None of the participants had any previous experience with neurostimulation techniques.

**Table 1:** Demographic characteristics of participants by their primary headache syndrome

			Episodic	Chronic	Tension-Type	Episodic	Chronic	Cluster	Episodic	Chronic
	All	Migraine	Migraine	Migraine	Headache (TTH)	TTH	TTH	Headache (CH)	CH	CH
	N (%)	N (%)	N (%)	N (%)	N (%)	N (%)	N (%)	N (%)	N (%)	N (%)
	514	372 (72.4)	220 (42.8)	152 (29.57)	107 (20.8)	25 (4.86)	82 (15.95)	35 (6.8)	14 (2.72)	21 (4.09)
Sex										
Male	132 (25.7)	69 (18.5)	47 (21.4)	22 (14.5)	38 (35.5)	8 (32.0)	30 (36.6)	25 (71.4)	12 (85.7)	13 (61.9)
Female	382 (74.3)	303 (81.5)	173 (78.6)	130 (85.5)	69 (64.5)	17 (68.0)	52 (63.4)	10 (28.6)	2 (14.3)	8 (38.1)
Age										
Mean (±SD)	41.71 (12.04)	40.65 (11.68)	38.17 (10.97)	44.23 (11.76)	44.65 (13.18)	42.60 (12.65)	45.28 (13.28)	43.97 (10.16)	39.14 (7.0)	47.19 (10.65)
< 20 yrs	8 (1.6)	7 (1.9)	6 (2.7)	1 (0.7)	1 (0.9)	0	1 (1.2)	0	0	0
20–39 yrs	229 (44.6)	180 (48.4)	126 (57.3)	54 (35.5)	37 (34.6)	11 (44.0)	26 (31.7)	12 (34.3)	7 (50.0)	5 (23.8)
40–60 yrs	236 (45.9)	162 (43.6)	80 (36.4)	82 (53.9)	54 (50.5)	12 (48.0)	42 (51.2)	20 (57.1)	7 (50.0)	13 (61.9)
> 60 yrs	41 (8.0)	23 (6.2)	8 (3.6)	15 (9.9)	15 (14.0)	2 (8.00)	13 (15.9)	3 (8.6)	0	3 (14.3)
Education										
1	13 (2.5)	8 (2.1)	2 (0.9)	6 (3.9)	5 (4.7)	1 (4.0)	4 (4.9)	0	0	0
2	24 (4.7)	17 (4.6)	10 (4.5)	7 (4.6)	5 (4.7)	0	5 (6.1)	2 (5.7)	0	2 (9.5)
3	178 (34.6)	126 (33.9)	86 (39.1)	40 (26.3)	39 (36.5)	7 (28.0)	32 (39.0)	13 (37.1)	4 (28.6)	9 (42.9)
4	14 (2.7)	12 (3.2)	8 (3.6)	4 (2.6)	2 (1.9)	1 (4.0)	1 (1.2)	0	0	0
5	21 (4.1)	14 (3.8)	8 (3.6)	6 (3.9)	5 (4.7)	2 (8.0)	3 (3.7)	2 (5.7)	0	2 (9.5)
6	231 (44.9)	178 (47.9)	94 (42.7)	84 (55.3)	40 (37.4)	14 (56.0)	26 (31.7)	13 (37.1)	6 (42.9)	7 (33.3)
7	28 (5.4)	16 (4.3)	11 (5.00)	5 (3.3)	8 (7.5)	0	8 (9.8)	4 (11.4)	4 (28.6)	0
8	5 (0.97)	1 (0.3)	1 (0.5)	0	3 (2.8)	0	3 (3.7)	1 (2.9)	0	1 (4.8)
Profession										
0	92 (17.9)	76 (20.4)	51 (23.2)	25 (16.4)	13 (12.1)	3 (12.0)	10 (12.2)	3 (8.6)	1 (7.1)	2 (9.5)
1	124 (24.1)	87 (23.4)	45 (20.5)	42 (27.6)	24 (22.4)	5 (20.0)	19 (23.2)	13 (37.1)	8 (57.1)	5 (23.8)
2	39 (7.6)	26 (7.0)	12 (5.5)	14 (9.2)	10 (9.4)	2 (8.0)	8 (9.8)	3 (8.6)	1 (7.1)	2 (9.5)
3	48 (9.3)	35 (9.4)	22 (10.0)	13 (8.6)	9 (8.4)	1 (4.0)	8 (9.8)	4 (11.4)	0	4 (19.0)
4	109 (21.2)	86 (23.1)	58 (26.4)	28 (18.4)	17 (15.9)	6 (24.0)	11 (13.4)	6 (17.1)	2 (14.3)	4 (19.0)
5	9 (1.8)	6 (1.6)	4 (1.8)	2 (1.3)	2 (1.9)	1 (4.0)	1 (1.2)	1 (2.9)	0	1 (4.8)
6	5 (1.0)	1 (0.3)	1 (0.5)	0	3 (2.8)	2 (8.0)	1 (1.2)	1 (2.9)	1 (7.)	0
7	4 (0.0)	2 (0.5)	1 (0.5)	1 (0.7)	2 (1.9)	0	2 (2.4)	0	0	0
8	9 (1.8)	7 (1.9)	4 (1.8)	3 (2.0)	2 (1.9)	1 (4.0)	1 (1.2)	0	0	0
9	26 (5.1)	19 (5.1)	11 (5.0)	8 (5.3)	4 (3.7)	1 (4.0)	3 (3.7)	3 (8.6)	1 (7.1)	2 (9.5)
10	49 (9.5)	27 (7.3)	11 (5.0)	16 (10.5)	21 (19.6)	3 (12.0)	18 (22.0)	1 (2.9)	0	1 (4.8)
Years with headaches										
< 3 yrs	86 (16.7)	48 (12.9)	36 (16.4)	12 (7.9)	34 (31.8)	11 (44.0)	23 (28.0)	4 (11.4)	4 (28.6)	0
3–5 yrs	66 (12.8)	39 (10.5)	33 (15.0)	6 (3.9)	19 (17.8)	6 (24.0)	13 (15.9)	8 (22.9)	2 (14.3)	6 (28.6)
6-10 yrs	106 (20.6)	79 (21.2)	48 (21.8)	31 (20.4)	17 (15.9)	4 (16.0)	13 (15.9)	10 (28.6)	4 (28.6)	6 (28.6)
> 10 yrs	256 (49.8)	206 (55.4)	103 (46.8)	103 (67.8)	37 (34.6)	4 (16.0)	33 (40.2)	13 (37.1)	4 (28.6)	9 (42.9)
Nocebo										
> 15	291 (56.6)	217 (58.3)	142 (64.5)	75 (49.3)	55 (51.4)	16 (64.0)	39 (47.6)	19 (54.3)	9 (64.3)	10 (47.6)
< 15	223 (43.4)	155 (41.7)	78 (35.4)	77 (50.7)	52 (48.6)	9 (36.0)	43 (52.4)	16 (45.7)	5 (35.7)	11 (52.4)

The education of participants classified according to the International Standard Classification of (ISCDE 2011) [11]: 1 = primary; 2 = lower secondary; 3 = upper secondary; 4 = post secondary non-tertiary; 5 = short-cycle tertiary; 6 = Bachelor’s or equivalent level; 7 = Master’s or equivalent level; 8 = Doctoral or equivalent level

The participant professions classified according to the International Standard Classification of Occupations (ISCO-08) [10]: 0 = unemployed, 1 = professionals, technicians and associate professionals, 2 = executive, administrative and managerial occupations; 3 = service and sales workers; 4 = administrative supporters; 5 = precision, production, craft and repair; 6 = machine and operators; 7 = transportation; 8 = handlers, cleaners, helpers and laborers; 9 = service occupations; and 10 = retired

**Table 2:** Participants’ preferences for headache treatment by primary headache disorder

				Episodic	Chronic	Tension-Type	Episodic	Chronic	Cluster	Episodic	Chronic
		All	Migraine	Migraine	Migraine	Headache (TTH)	TTH	TTH	Headache (CH)	CH	CH
Q No	Question	N (%)	N (%)	N (%)	N (%)	N (%)	N (%)	N (%)	N (%)	N (%)	N (%)
	Symptomatic treatment										
Q4	More important is:										
	Safety	88 (17.1)	60 (16.1)	38 (17.3)	22 (14.5)	22 (20.8)	7 (28.0)	15 (18.5)	6 (17.1)	4 (28.6)	2 (9.5)
	Efficacy	416 (80.9)	308 (82.8)	180 (81.8)	128 (84.2)	81 (76.4)	17 (68.0)	64 (79.0)	27 (77.1)	10 (71.4)	17 (81.0)
	Route of administration	9 (1.8)	4 (1.1)	2 (0.9)	2 (1.3)	3 (2.8)	1 (4.0)	2 (2.5)	2 (5.7)	0	2 (9.5)
Q5	They prefer to take:										
	A pill	452 (88.1)	327 (87.9)	190 (86.4)	137 (90.1)	95 (89.6)	21 (84.0)	74 (91.4)	30 (85.7)	10 (71.4)	20 (95.2)
	A suppository	17 (3.3)	14 (3.8)	10 (4.5)	4 (2.6)	2 (1.9)	0	2 (2.5)	1 (2.9)	0	1 (4.8)
	A nasal sprey	27 (5.3)	18 (4.8)	14 (6.4)	4 (2.6)	5 (4.7)	4 (16.0)	1 (1.2)	4 (11.4)	4 (28.6)	0
	sc injection	4 (0.8)	4 (1.1)	2 (0.9)	2 (1.3)	0	0	0	0	0	0
	Im injection	9 (1.8)	5 (1.3)	3 (1.4)	2 (1.3)	4 (3.8)	0	4 (4.9)	0	0	0
	iv injection	4 (0.8)	4 (1.1)	1 (0.5)	3 (2.0)	0	0	0	0	0	0
Q6	They do not want to take:										
	Pill	11 (2.2)	6 (1.6)	6 (2.7)	0	4 (3.8)	0	4 (4.9)	1 (2.9)	1 (7.1)	0
	Suppository	148 (29.0)	102 (27.6)	53 (24.1)	49 (32.7)	37 (34.9)	8 (32.0)	29 (35.8)	9 (25.7)	3 (21.4)	6 (28.6)
	Nasal sprey	35 (6.8)	30 (8.1)	14 (6.4)	16 (10.7)	3 (2.8)	0	3 (3.7)	2 (5.7)	0	2 (9.5)
	sc injection	18 (3.5)	12 (3.2)	11 (5.0)	1 (0.7)	3 (2.8)	3 (12.0)	0	3 (8.6)	1 (7.1)	2 (9.5)
	Im injection	70 (13.7)	54 (14.6)	35 (15.9)	19 (12.7)	13 (12.3)	3 (12.0)	10 (12.3)	3 (8.6)	1 (7.1)	2 (9.5)
	iv injection	229 (44.8)	166 (44.9)	101 (45.9)	65 43.3)	46 (43.4)	11 (44.0)	35 (43.2)	17 (48.6)	8 (57.1)	9 (42.9)
Q7	They prefer more:										
	Drugs	168 (32.7)	120 (32.3)	83 (37.7)	37 (24.5)	37 (34.6)	7 (28.0)	30 (36.6)	11 (31.4)	5 (35.7)	6 (28.6)
	A device	345 (67.3)	251 (67.7)	137 (62.3)	114 (75.5)	70 (65.4)	18 (72.0)	52 (63.4)	24 (68.6)	9 (4.3)	15 (71.4)
	Preventive treatment										
Q8	More important is:										
	Safety	124 (24.1)	86 (23.1)	53 (24.1)	33 (21.7)	30 (28.0)	8 (32.0)	22 (26.8)	8 (22.9)	6 (42.9)	2 (9.5)
	Efficacy	372 (72.4)	271 (72.8)	158 (71.8)	113 (74.3)	74 (69.2)	16 (64.0)	58 (70.7)	27 (77.1)	8 (57.1)	19 (90.5)
	Route of administration	18 (3.5)	15 (4.0)	9 (4.1)	6 (3.9)	3 (2.80	1 (4.0)	2 (2.4)	0	0	0
Q9	They prefer to take:										
	1 pill/day	275 (53.8)	189 (51.1)	122 (55.5)	67 (44.7)	67 (63.2)	16 (64.0)	51 (63.0)	19 (54.3)	7 (50.0)	12 (57.1)
	2 pills/ day	39 (7.6)	27 (7.3)	18 (8.2)	9 (6.0)	9 (8.5)	4 (16.0)	5 (6.2)	3 (8.6)	2 (14.3)	1 (4.8)
	3 pills/day	10 (2.0)	8 (2.2)	6 (2.7)	2 (1.3)	0	0	0	2 (5.7)	0	2 (9.5)
	sc injection/ month	41 (8.0)	30 (8.1)	22 (10.0)	8 (5.3)	9 (8.5)	2 (8.0)	7 (8.6)	2 (5.7)	0	2 (9.5)
	iv injection/ month	16 (3.1)	14 (3.8)	9 (4.1)	5 (3.3)	1 (0.9)	0	1 (1.2)	1 (2.9)	1 (7.1)	0
	sc injection/ 3 month	68 (13.3)	55 (14.9)	27 (12.3)	28 (18.7)	9 (8.5)	0	9 (11.1)	4 (11.4)	2 (14.3)	2 (9.5)
	iv injection/ 3 month	62 (12.1)	47 (12.7)	16 (7.3)	31 (20.7)	11 (10.4)	3 (12.0)	8 (9.9)	4 (11.4)	2 (14.3)	2 (9.5)
Q10	They do not want to take:										
	1 pill/day	5 (1.0)	3 (0.8)	3 (1.4)	0	2 (1.9)	0	2 (2.5)	0	0	0
	2 pills/ day	7 (1.4)	5 (1.4)	4 (1.8)	1 (0.7)	2 (1.9)	1 (4.0)	1 (1.2)	0	0	0
	3 pills/day	163 (32.0)	122 (33.1)	60 (27.3)	62 (41.6)	32 (30.2)	8 (32.0)	24 (29.6)	9 (25.7)	3 (21.4)	6 (28.6)
	sc injection/ month	67 (13.1)	44 (11.9)	26 (1.8)	18 (12.1)	18 (17.0)	3 (12.0)	15 (18.5)	5 (14.3)	2 (14.3)	3 (14.3)
	iv injection/ month	196 (38.4)	146 (39.6)	101 (45.9)	45 (30.2)	42 (39.6)	11 (44.0)	31 (38.3)	8 (22.9)	3 (21.4)	5 (23.8)
	sc injection/ 3 month	15 (2.9)	9 (2.4)	5 (2.3)	4 (2.7)	1 (0.9)	0	1 (1.2)	5 (14.3)	2 (14.3)	3 (14.3)
	iv injection/ 3 month	57 (11.2)	40 (10.8)	21 (9.5)	19 (12.8)	9 (8.5)	2 (8.0)	7 (8.6)	8 (22.9)	4 (28.6)	4 (19.0)
Q11	They prefer more:										
	Drugs	189 (36.9)	136 (36.7)	96 (43.6)	40 (26.5)	43 (40.6)	8 (32.0)	35 (43.2)	10 (28.6)	3 (21.4)	7 (33.3)
	A device	323 (63.1)	235 (63.3)	124 (56.4)	111 (73.5)	63 (59.4)	17 (68.0)	46 (56.8)	25 (71.4)	11 (78.6)	14 (66.7)

For questions see Appendix

Most participants (80.9%) judged that the efficacy is more important than the safety or the route of administration of a symptomatic treatment for headache; the large majority (88.1%) preferred the oral than other routes of administration for the drugs; interestingly, they also preferred neurostimulation instead of any pharmaceutical treatment (67.3%). More participants (72.4%) rated that the efficacy is more important than the safety or the route of administration of a treatment for the prevention of headache disorders; they choose (53.8%) a pill once daily than other routes of drug administration (including monthly subcutaneous or intravenous injections); like for symptomatic treatment, they preferred an external device for neurostimulation (63.1%) instead of any pharmaceutical prophylactic treatment. Two hundred ninety one participants (56.6%) scored more than 15 on the Q-No questionnaire, indicating potential nocebo behaviors (Table 1).

### Subgroup analyses

Participants’ preferences for the preventive headache treatment varied by the frequency of headache attacks they were suffering from. Those they were suffered from chronic headache disorders reported more often that they preferred neurostimulation than daily pharmaceutical treatment versus those they were suffered from episodic headache disorders (OR = 1.5, 95% CI:[1.1–2.1]; *p* = 0.013). Among several types of primary headache disorders those participants they were suffered from chronic migraine reported more often as well that they preferred an external neurostimulation device than any pharmaceutical treatment for migraine prophylaxis versus those they were suffered from any other primary headache disorders (OR = 2.15, 95% CI:[1.4–3.4]; *p* < 0.01).

Those participants they scored more than 15 in the Q-No questionnaire they preferred to use daily external neurostimulation than daily drug treatment (OR = 1.6, 95% CI:[1.1–2.3]; *p* < 0.05) for headache prevention. They also prefer to use acute neurostimulation for symptomatic headache treatment than drugs (OR = 1.7, 95%CI: [1.1–2.5], *p* = 0.008).

Statistics did not reveal any other differences in patients’ preferences including analyses for gender, age, occupation and education (data not shown).

## Discussion

In this survey the patients’ preferences for headache symptomatic and preventive treatment have been recorded. Almost four out of five headache sufferers reported that they cared for more efficacy than for the safety or route of administration of the symptomatic or preventive treatments they were taking. Although naïve to neurostimulation and to new injectable anti-migraine treatments, two out of three patients preferred to use an external neurostimulator rather a drug to treat their headaches, both acutely or prophylactically (including the injectable agents every month). More than one of two patients preferred to take a pill once a day than an injection once a month or every three months for pharmaceutical prevention, assuming that all treatments have a comparable efficacy and safety profile. The type of headache the patients were suffered from did not affect their choices with one exception: those they were suffered from chronic headaches and from chronic migraine reported more often that they preferred an external neurostimulation device for acute and prophylactic treatment. An external device was significantly more preferable among patients with potential nocebo behaviors compared to those with low risk for nocebo as well.

Therefore it appears that headache patients insist to prefer and trust the traditional ways of treatments among drugs (a pill to be taken orally once a day). As someone may expect they do not want to take a pill many times per day for prophylactic treatment, nor get an injection for acute headache treatment. On the other hand, they indicated a clear preference favoring external neurostimulation, although naïve to this technique. What causes this preference remains unclear from the study data. Because it was declared that efficacy and safety are hypothetically equal between treatments it cannot be assumed that safety issues are hiding behind this choice. Yet safety stands a major issue for a chronic treatment. Nor life style reasons can explain this preference as well. A positive expectation favoring an entire novel treatment driven from outside the human body could serve as a potential explanation but further investigation towards this direction is needed.

No other study has been conducted to record patient preferences related to neurostimulation in headache and pain versus traditional pharmaceutical treatments. There are only a few studies published the last decade investigating patients’ preferences for headache treatment, most of them focused on which drug category the patients may prefer [5, 10–17]. In one study that investigated patients’ preferences for migraine prevention, the patients rated efficacy as the most important aspect in preventive therapy and preferred treatment options with higher efficacy rates [15], like in the present study. In another prospective study, patients changed their preferences favoring a nasal formulation of zolmitriptan because of the speed of onset and the overall efficacy compared to conventional zolmitritpan tablets [14], again indicating that efficacy matters most for the symptomatic treatment. Therefore, in all studies performed including the present one, headache sufferers rate the efficacy as the most important aspect of the treatment. In addition, the participants of this study did not like to be treated intravenously either acutely for symptomatic treatment or repetitively for prophylaxis. The patients’ choices recorded here might predict a limited preference for the use of the novel injectable prophylactic treatments for migraine and cluster headache with monoclonal anti-bodies. The anecdotal enthusiastic participation in the clinical trials for these novel treatments (both for injectable and neurostimulation) across Europe including Greece may contradict with our results however.

Two out of five participants scored more than 15 in the Q-No questionnaire indicating potential nocebo behaviors. In meta-analyses for nocebo in clinical trials, eight out of 20 patients treated with placebo experienced any adverse event. More importantly, one out of 20 patients treated with placebo withdrew treatment because of adverse events. The adverse events in placebo groups mirrored the adverse events expected of the active medication studied, confirming that pretrial suggestions induce the adverse events in placebo-treated patients. Nocebo was higher in preventive treatments than in symptomatic ones [18, 19]. This is the first report of real life data using the Q-No questionnaire [8], showing that one out of two headache sufferers are in high risk to express nocebo behaviors resulting in limited adherence. Primary headache disorders are usually treatable but due to safety and tolerability reasons, available preventive treatments have often limited success, even in the right hands [4]. There is no doubt today that some of those headache sufferers, who will discontinue the treatment because of safety or tolerability, are powered by nocebo [18, 19]. Among other co-factors, patients’ negative expectation and previous unpleasant treatment experiences create negative believes for the treatment outcome and safety, generating nocebo. Physicians treating headache sufferers should acknowledge nocebo as a significant cofactor for treatment adherence and failure and plan techniques to border nocebo, such as patients’ education and close follow-up. Positive suggestions and continuous support increase patient’s compliance and decreases nocebo.

### Study strengths and limitations

This is the first study reporting patient preferences related to headache treatment options that include external neurostimulation and monthly injectable agents that are under investigation for migraine and cluster headache treatment. A large proportion of headache sufferers display nocebo behaviors that may affect these preferences. There are several limitations however. Participants had not experienced these new treatments that are still under investigation and results of phase 3 trials are missing to better compare their efficacy and long term safety; the size population; and patient selection biases including cultural ones. Thus, the results may not be completely generalizable to other practices.

### Suggestions for further targeted research

In future studies, subgroup analysis could be performed to determine whether prior experience with both neurostimulation and monoclonal antibodies, or the presence of depressive or anxiety symptoms impacts the congruence between patient expectations and actual practice regarding decision making at the time of a treatment prescription.

## Conclusion

Headache sufferers prefer the external neurostimulation rather than the pharmaceutical treatment for their headaches, those who suffer from chronic headaches and chronic migraine in particular. A large proportion of headache sufferers have noticed nocebo behaviors that may control their treatment choices. In the light of several novel up-coming treatments these patient preferences are important for clinicians, insurances and health policy makers.

## Clinical implications

- Headache sufferers prefer to use an external device to treat their headaches, both for symptomatic and preventive treatment.
- Regardless of the primary headache disorder they suffer from, patients prefer to use a pill once daily to prevent their headaches rather an injection once a month or every three months.
- Nocebo is very prevalent among headache sufferers and may affect their choices for the treatment.
- Headache health providers should explore personal patients’ preferences before treatment decision-making and manage potential nocebo behaviors.

## Appendix

### Hellenic headasche society

#### A questionnaire to record the preferences of patients seeking neurological consultation because of headache

Headaches is the third cause of life years lost because of disability in general population world-wide; so you are not alone, many of other people are suffering from headaches like you. This questionnaire is anonymous and aims to make physicians to better understand how patients prefer to take a headache treatment. In other words, which issues are more important for you when your doctor suggests a treatment for your headaches? Patients’ opinion is significant for designing new treatments that are not necessary pharmaceuticals. The results of this survey that is covered by the auspices of the HELLENIC HEADACHE SOCIETY will be presented in Medical Congresses and will be published in Scientific Journals. Please read carefully the questions below and answer. It will take only 5 min from your time.

Please report.

Date of birth, sex, education, profession.

**Q1.** Since when do you suffer from headaches (please note year and month)?

**Q2.** Since when your headaches have being became daily (please note year and month)?

**Q3.** Approximately, how many days during a month do you have any kind of headache?

#### The following questions are related to the symptomatic-acute treatment you are taking for your headache attacks

**Q4.** What is more important for a pharmaceutical treatment you are taking to treat/stop a headache attack (please mark only one answer)?

1. The safety of drug treatment, if there are adverse events and what impact they have in me.
2. The efficacy of the drug treatment, if my headaches will be cured or improved.
3. The route of drug administration, if the drug is a pill or injection or a supposition, etc.

**Q5.** Which route of drug administration you prefer most (please mark only one answer)?

1. Via the mouth
2. Via the rectal
3. Via the nose
4. Via the skin (injection)
5. Via the muscle (injection in muscles)
6. Via the vein (injection in the vein)

**Q6.** In the previous question please mark, which route you unlike most.

1. Via the mouth
2. Via the rectal
3. Via the nose
4. Via the skin (injection)
5. Via the muscle (injection in muscles)
6. Via the vein (injection in the vein)

**Q7.** There are novel treatments with neurostimulation devices. Meaning you will use this external device to cure your headache attack with an electrical of magnetic stimulation. What do you prefer most?

1. A drug.
2. A neurostimulation device.

#### The following questions are related to the preventive treatment you are taking daily to decrease the frequency of your hedache attacks

**Q8.** What is more important for a pharmaceutical treatment you are taking daily to make your headaches become more rare/scarce and less severe (please mark only one answer)?

1. The safety of drug treatment, if there are adverse events and what impact they have in me.
2. The efficacy of the drug treatment, if my headaches will be cured or improved.
3. The route of drug administration, if the drug is a pill or injection or a suppository, etc.

**Q9.** Which route of drug administration do you prefer most, independently to safety and efficacy? (Consider that all drugs share the same efficacy and safety and please mark only one answer)

1. Via the mouth
2. Via the rectal
3. Via the nose
4. Via the skin (injection)
5. Via the muscle (injection in muscles)
6. Via the vein (injection in the vein)

**Q10.** In the previous question please mark, which route you unlike most.

1. Via the mouth
2. Via the rectal
3. Via the nose
4. Via the skin (injection)
5. Via the muscle (injection in muscles)
6. Via the vein (injection in the vein)

**Q11.** There are novel treatments with neurostimulation devices. Meaning you will use this external device that delivers electrical or magnetic stimulation daily to make your headache scarce and less severe. What do you prefer most?

1. A drug.
2. A neurostimulation device.

THANK YOU for taking the time to participate in this survey that aims to improve health services related to headaches.

### Hellenic headache society

#### Physician’s section

Headache diagnosis, Years with headaches, months with preventive treatment (please specify the treatments), months with symptomatic treatment (please specify the treatments), medication overuse, other concomitant medical condition.
